# Investigating the effect of COVID-19 dissemination on symptoms of anxiety and depression among university students

**DOI:** 10.1192/bjo.2021.24

**Published:** 2021-03-19

**Authors:** Daniel Vigo, Laura Jones, Richard Munthali, Julia Pei, Jean Westenberg, Lonna Munro, Carolina Judkowicz, Angel Y. Wang, Brianna Van den Adel, Joshun Dulai, Michael Krausz, Randy P. Auerbach, Ronny Bruffaerts, Lakshmi Yatham, Anne Gadermann, Brian Rush, Hui Xie, Krishna Pendakur, Chris Richardson

**Affiliations:** Department of Psychiatry, University of British Columbia, Canada; Department of Psychiatry, University of British Columbia, Canada; Department of Psychiatry, University of British Columbia, Canada; Department of Psychiatry, University of British Columbia, Canada; Department of Psychiatry, University of British Columbia, Canada; Department of Psychiatry, University of British Columbia, Canada; Department of Psychiatry, University of British Columbia, Canada; Department of Psychiatry, University of British Columbia, Canada; Department of Psychiatry, University of British Columbia, Canada; Department of Psychiatry, University of British Columbia, Canada; Department of Psychiatry, University of British Columbia, Canada; Department of Psychiatry, Columbia University, New York, USA; Center for Public Health Psychiatry, Universitair Psychiatrisch Centrum KU Leuven, Belgium; Department of Psychiatry, University of British Columbia, Canada; School of Population and Public Health, University of British Columbia, Canada; Department of Psychiatry & School of Public Health, University of Toronto, Canada; Faculty of Health Sciences, Simon Fraser University, Canada; Department of Economics, Simon Fraser University, Canada; School of Population and Public Health, University of British Columbia, Canada

**Keywords:** Psychiatric epidemiology, COVID-19, anxiety disorders, depressive disorders, student population

## Abstract

**Background:**

Evidence about the impact of the COVID-19 pandemic on the mental health of specific subpopulations, such as university students, is needed as communities prepare for future waves.

**Aims:**

To study the association of proximity of COVID-19 with symptoms of anxiety and depression in university students.

**Method:**

This trend study analysed weekly cross-sectional surveys of probabilistic samples of students from the University of British Columbia for 13 weeks, through the first wave of COVID-19. The main variable assessed was propinquity of COVID-19, defined as ‘knowing someone who tested positive for COVID-19’, which was specified at different levels: knowing someone anywhere globally, in Canada, in Vancouver, in their course or at home. Proximity was included in multivariable linear regressions to assess its association with primary outcomes, including 30-day symptoms of anxiety and/or depression.

**Results:**

Of 1388 respondents (adjusted response rate of 50%), 5.6% knew someone with COVID-19 in Vancouver, 0.8% in their course and 0.3% at home. Ten percent were overwhelmed and unable to access help. Knowing someone in Vancouver was associated with an 11-percentage-point increase in the probability of 30-day anxiety symptoms (s.e. 0.05, *P* ≤ 0.05), moderated by gender, with a significant interaction of the exposure and being female (coefficient −20, s.e. 0.09, *P* ≤ 0.05). No association was found with depressive symptoms.

**Conclusions:**

Propinquity of COVID-19 cases may increase the likelihood of anxiety symptoms in students, particularly among men. Most students reported coping well, but additional support is needed for an emotionally overwhelmed minority who report being unable to access help.

COVID-19 and the measures introduced to prevent its spread have upended daily lives globally. Given its multisystemic nature and the impact of pervasive restrictions on physical, social and economic activities, adverse mental health outcomes are expected.^1–4^ Increased rates of anxiety and depression have been reported among front-line health workers, migrant workers, the elderly and young adults, particularly university students.^5–13^ Although these emerging data are informative, they must be interpreted with caution because many studies have used non-probabilistic sampling, which results in insufficient representativeness and generalisability.^14^

## Disruption of student life due to COVID-19

A more granular understanding of the impact of the first wave of COVID-19 on different subpopulations is relevant to manage future restrictions.^15,16^ Universities and colleges play key societal roles by training a professional and academic workforce that contributes to scientific progress, technological innovation and economic growth. They also play a stewardship role with transitional-age youth, as they move from adolescence and the nuclear family to adulthood and public life. In the context of COVID-19, many institutions are foregoing in-person education for the autumn term.^17,18^ Although the risks of resuming in-person education are clear, the externalities of universities going online are not fully understood, including effects on organisations, faculty, staff and students.^11,19–23^ Common mental health-related symptoms, most notably anxiety and depression, are pervasive among university students, and the stressors associated with the pandemic can be expected to exacerbate adverse outcomes.^12,24–28^ During the pandemic, feelings of fear and worry about one's own health and that of their loved ones, trouble concentrating, disruptions to sleeping patterns, decreased social interactions and increased concerns on academic performance have been frequently reported by students as contributors to increased levels of stress, anxiety and depressive thoughts.^29^ Alarmingly, in a cross-sectional survey study of over 2000 USA college students, almost 50% reported moderate-to-severe levels of depression, close to 40% reported moderate-to-severe levels of anxiety and a fifth reported having suicidal thoughts during the COVID-19 pandemic.^30^ The drop in overall mental health among students has been associated with an observed shift in students’ priorities, from academics toward health- and financial-related worries.^31^ Also, disrupted mental health services may compound the effects of the pandemic.^32,33^ In-person services and peer support groups have been cancelled or have been heavily restricting capacity, and support by telephone or online can be challenging and disengaging for students.^34,35^

To make rational public health decisions, knowledge of the effects of the first wave of COVID-19 is needed. We examined the dissemination of COVID-19 on a large university campus in British Columbia, Canada, focussing on how symptoms of anxiety and depression evolved during part of the 2020 winter term (February to May), as the first wave spread globally, reached campus, peaked and receded. We aimed to explore the association of geographic and social proximity of COVID-19 cases with the probability of manifesting symptoms of anxiety and/or depression, and understand how COVID-19 affects the general student population (which includes, but is not restricted to, those that meet criteria for a disorder). We also sought to understand whether specific population characteristics interact with COVID-19 in its association with these symptoms.

## Method

### Study design

This trend study is based on data collected through repeated cross-sectional deployment of the World Health Organization (WHO) World Mental Health-International College Student (WMH-ICS) survey. The WMH-ICS survey is a self-administered interview based on the WHO World Mental Health-Composite International Diagnostic Interview (WHO WMH-CIDI), and is part of the WHO World Mental Health Survey Initiative.^36,37^ The WMH-ICS initiative has been developed to help coordinate epidemiological research among college students worldwide, and has been repeatedly used in the literature.^38–42^ It uses validated screening instruments to generate estimates of the presence of a wide range of mental disorders, coupled with probe questions to evaluate symptom severity, help-seeking behaviour and other episode-related questions.^36^

The survey was originally implemented to capture variations in symptoms in response to predictable and unpredictable stressors throughout the year, such as examinations, graduation and natural and human-made disasters. In the first week of March 2020 (week 4 of the survey), as COVID-19 emerged as an exposure of interest, questions were included to assess weekly variations in the social and geographic proximity of COVID-19. In week 9, an additional question was included inquiring whether respondents felt emotionally affected or overwhelmed, and whether they had been able to access help (see Supplementary Appendix 1, Box 1 available at https://doi.org/10.1192/bjo.2021.24). Knowing someone who tested positive for COVID-19 represented the exposure of interest.

### Outcome measures

Our primary outcome measures were derived from the WHO WMH-CIDI screening questions assessing 30-day anxiety (four items) and depression (four items) (see Supplementary Appendix 1, Box 2). These items were selected to capture the experience of symptoms of anxiety and depression. Symptoms of anxiety and depression were assessed via Likert-type responses to how often respondents had any such symptoms during the past 30 days. Response options were ‘none of the time’, ‘a little of the time’, ‘some of the time’, ‘most of the time’ and ‘all or almost all of the time’. Participants who responded ‘some of the time’ or higher to at least one item were considered to have screened positive for experiencing ‘anxiety or depression symptoms’, ‘anxiety symptoms’ and/or ‘depression symptoms’.

### Procedure

This study covers 13 weeks of the term that ended in May 2020, roughly coinciding with the first wave of COVID-19 in Vancouver, Canada. Each week, the survey was sent to a new sample of 350 students generated via stratified random sampling (by programme, year, international student status, gender and age) from all currently enrolled students. For each of the 13 weeks, the initial recruitment of 350 students occurred by invitation and reminder emails. Ten days after sending the initial invitation email, a second phase recruitment procedure then followed up with a random subsample of 70 non-responders with telephone calls, text messages or personal email invitations to participate as per a specified protocol for ‘hard to reach’ and ‘very hard to reach’ students (see Supplementary Appendix 1, Box 3). The goal of the assertive follow-up was to increase response rate by diversifying the outreach method and explaining the importance of getting responses from initial non-responders. The adjusted response rate was 50%, using the American Association for Public Opinion research weighted response rate 1 (RR1w) calculation for two-phase sample designs.^43^

Supplementary Appendix 2 contains a table with respondent characteristics in the different subsamples, and the methodology for obtaining the RR1w (Supplementary Table 4 and Box 1).

### Ethics

The authors assert that all procedures contributing to this work comply with the ethical standards of the relevant national and institutional committees on human experimentation and with the Helsinki Declaration of 1975, as revised in 2008. All procedures involving human subjects were approved by Behavioural Research Ethics Board of the University of British Columbia (approval number H19-02538). Written informed consent was obtained from all participants before completing the WMH-ICS survey.

### Statistical analyses

First, we present descriptive findings and the gradual increase in social and geographic proximity of COVID-19 to campus. Chi-squared and Fisher's exact tests were used to identify differences in anxiety and/or depression outcomes across groups defined by sociodemographic characteristics and pre-existing mental health conditions.

Second, we show the percentage of students that felt emotionally unaffected, affected or overwhelmed by the pandemic, as well as whether they were able to manage or find help.

Third, to examine the association between the primary exposure of interest (i.e. knowing someone who tested positive for COVID-19) and each of the mental health outcomes (30-day symptoms of anxiety or depression, 30-day symptoms of anxiety, 30-day symptoms of depression), we ran separate univariable linear regressions with the exposure and other relevant variables. We then ran three separate multivariable linear regressions (one for each outcome) including all covariates: gender, age, lifetime symptoms of anxiety, lifetime symptoms of depression, a set of dummy variables or indicators for completion week (an indicator of time), respondent type (easy versus hard versus very hard to reach), ethnicity, programme, programme year, international/Canadian student status and housing type. Finally, we ran three separate multivariable linear regressions, including interactions between the exposure and specific covariates that, based on clinical expertise, may moderate the associations of the exposure with the outcomes: gender, lifetime history of anxiety and/or depression symptoms, and survey completion week, which served as an indicator of time. For all analyses, a complete-case analysis approach was used and the cut-off for significance was set at 5% (*P* < 0.05). Multivariable linear regressions were used to yield absolute risk differences between exposed and unexposed groups, adjusting for covariates. When the outcome variable is dichotomous, it is common in the epidemiology literature to use logistic regression, rather than ordinary least squares (OLS), to estimate the effect of treatment variables on outcome variables. If the model is correctly specified, both approaches yield estimates that are unbiased as the sample size grows to infinity (i.e. they are consistent). However, if the model is incorrectly specified, estimates from logistic regression are hard to interpret. In contrast, if the model is incorrectly specified, the OLS estimator still retains a useful interpretation: it gives the best linear estimator of the conditional expectation function.^44^ For this reason, we use OLS regression throughout this paper. The OLS estimator we use corresponds to the ‘linear probability model’, where the conditional expectation of the outcome variable is the probability that it takes the value 1, conditional on a linear index of the covariates. Thus, we may interpret estimated coefficients as giving the marginal effect of a one-unit change in a covariate on the conditional probability that Y equals 1. The linear regressions were run using OLS estimation, with the robust s.e. VCE (robust) option to provide valid s.e., *P*-values and unbiased estimates. Qualtrics was used to administer the survey and data were analysed with Stata version 15.1 for Windows.^45^

## Results

Details of the distribution of the stratifying variables in respondents and in the general student population can be found in Supplementary Appendix 2, Table 4. Both populations are remarkably consistent, with the exception of a higher fraction of women (63% of respondents *v.* 56% of the general population), and a smaller fraction of non-degree students (1.5% *v.* 4.5%).

Data from 1388 respondents indicate that during the past 30 days, 61% endorsed symptoms of depression, 71% endorsed symptoms of anxiety and 78% endorsed symptoms of anxiety or depression ‘some of the time’ or more (Table 1). Further, our results show that there were statistically significant differences in 30-day symptoms of anxiety across gender, respondent type, lifetime symptoms of anxiety and/or depression, and knowing someone who tested positive for COVID-19 in general and in Vancouver. Statistically significant differences in 30-day symptoms of depression were found across groups defined by gender, lifetime symptoms of anxiety and/or depression, student year, ethnicity and housing type (Table 1).

**Table 1:** Study characteristics and subpopulations by anxiety symptoms, depression symptoms and anxiety or depression symptoms^a^

Variable	Anxiety, *n* (%)		Depression, *n* (%)		Anxiety or depression, *n* (%)	
	No	Yes	No	Yes	No	Yes
Gender		***		***		***
Male	178 (46.11)	283 (29.92)	220 (41.75)	251 (30.02)	137 (46.28)	332 (31.38)
Female	206 (53.37)	648 (68.50)	304 (57.69)	570 (68.18)	159 (53.72)	709 (67.01)
Other^b^	Removed	Removed	Removed	Removed	Removed	Removed
Survey type		*				
Initial survey (reference group)	299 (77.26)	791 (83.62)	421 (79.73)	697 (83.37)	230 (77.44)	879 (83.08)
Hard to reach with telephone	73 (18.86)	124 (13.11)	88 (16.67)	112 (13.40)	56 (18.86)	144 (13.61)
Hard to reach without telephone	15 (3.88)	31 (3.28)	19 (3.60)	27 (3.23)	11 (3.70)	35 (3.31)
History of depression?		***		***		***
No	244 (63.21)	325 (34.50)	320 (60.84)	265 (31.81)	200 (67.57)	378 (35.86)
Yes	142 (36.79)	617 (65.50)	206 (39.16)	568 (68.19)	96 (32.43)	676 (64.14)
History of anxiety?		***		***		***
No	166 (42.89)	132 (14.0)	185 (35.04)	117 (14.01)	133 (44.78)	166 (15.70)
Yes	221 (57.11)	813 (86.03)	343 (64.96)	718 (85.99)	164 (55.22)	891 (84.30)
Knows someone who tested positive for COVID-19?		*		*		*
No	350 (90.44)	811 (85.73)	473 (89.58)	716 (85.65)	270 (90.91)	912 (86.20)
Yes	37 (9.56)	135 (14.27)	55 (10.42)	120 (14.35)	27 (9.09)	146 (13.80)
Knows someone in Vancouver who tested positive for COVID-19?		*		*		**
No	375 (96.90)	885 (93.55)	507 (96.02)	782 (93.54)	291 (97.98)	991 (93.67)
Yes	12 (3.10)	61 (6.45)	21 (3.98)	54 (6.46)	6 (2.02)	67 (6.33)
Anyone with flu-like or respiratory symptoms in their classes? *n* = 988		*		*		*
No	256 (87.07)	564 (81.27)	344 (86.00)	492 (80.79)	198 (87.61)	633 (81.57)
Yes	38 (12.93)	130 (18.73)	56 (14.00)	117 (19.21)	28 (12.39)	143 (18.43)
Type of student				**		
First-year undergraduate	66 (17.60)	152 (16.78)	87 (17.13)	142 (17.71)	49 (17.13)	176 (17.32)
Second-year undergraduate	56 (14.93)	148 (16.34)	70 (13.78)	139 (17.33)	41 (14.34)	166 (16.34)
Third-year undergraduate	64 (17.07)	162 (17.88)	82 (16.14)	149 (18.58)	50 (17.48)	181 (17.81)
Fourth-year undergraduate	62 (16.53)	153 (16.89)	75 (14.76)	143 (17.83)	46 (16.08)	172 (16.93)
Graduate	91 (24.27)	229 (25.28)	140 (27.56)	184 (22.94)	72 (25.17)	250 (24.61)
Other	36 (9.60)	62 (6.84)	54 (10.63)	45 (5.61)	28 (9.79)	71 (6.99)
Ethnicity				*		
White	120 (31.09)	341 (36.08)	192 (36.43)	275 (32.93)	96 (32.43)	371 (35.10)
First Nations, Inuit or Metis	8 (2.07)	24 (2.54)	12 (2.28)	21 (2.51)	5 (1.69)	28 (2.65)
Chinese	128 (33.16)	269 (28.47)	171 (32.45)	233 (27.90)	102 (34.46)	298 (28.19)
Other minority	130 (33.68)	311 (32.91)	152 (28.84)	306 (36.65)	93 (31.42)	360 (34.06)
Housing type				*		
With parents or other relatives	103 (26.61)	257 (27.17)	121 (22.92)	249 (29.78)	73 (24.58)	296 (27.98)
In their own home or apartment	113 (29.20)	285 (30.13)	174 (32.95)	232 (27.75)	95 (31.99)	307 (29.02)
In a university owned or operated residence or fraternity	101 (26.10)	197 (20.82)	128 (24.24)	178 (21.29)	78 (26.26)	225 (21.27)
In a shared house, apartment or flat	65 (16.80)	196 (20.72)	98 (18.56)	168 (20.10)	47 (15.82)	218 (20.60)
Other	5 (1.29)	11 (1.16)	7 (1.33)	9 (1.08)	4 (1.35)	12 (1.13)

Total sample of 1388.

a. Only significant results shown, see complete table in Supplementary Appendix 2.

b. Cell contents have been removed because of small numbers.

* *P* ≤ 0.05, **P ≤ 0.01, ***P ≤ 0.001; both chi-squared and Fisher's exact tests were used where the cell counts are small.

### Geographic and social proximity of COVID-19

**Fig. 1:**
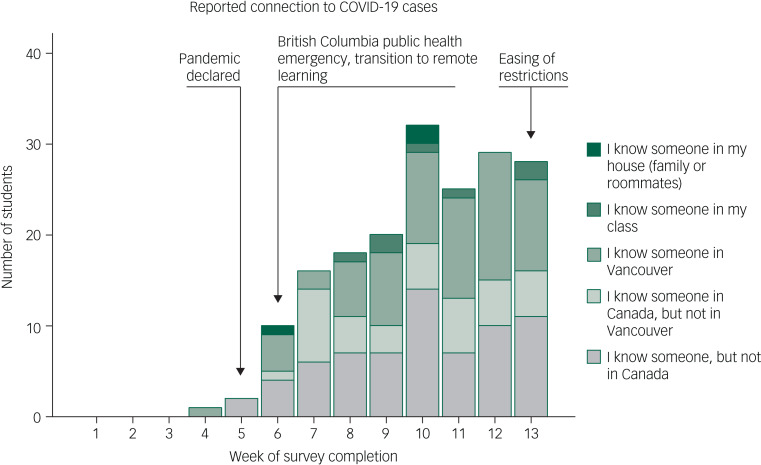
Number of participants reporting that they know someone with COVID-19.

Figure 1 shows the increase across time in proximity of COVID-19 to campus, and key population-level exposures: declaration of a global pandemic and of a local public health emergency, transition to remote learning and easing of restrictions. The survey was launched on 9 February 2020, COVID-19 questions were added on 2 March and an additional COVID-19 question was included on 9 April. There were three positive cases reported in the Vancouver catchment area in February, and ten cases during the first week of March (week 4).^46^ Of all the people who completed the survey during week 4 (after 13 cumulative cases), only one respondent knew someone with COVID-19 locally. Before week 1, there was one case in the Vancouver catchment area, zero cases during weeks 1 and 2, and two cases during week 3. Given that after 13 cumulative cases only one respondent knew someone with COVID-19, respondents from weeks 1 to 3 (during which there were three cumulative cases in Vancouver) were imputed ‘no’ for the ‘knows someone in Vancouver’ question.

Overall, 5.6% of respondents knew someone in Vancouver, 0.8% knew someone in their class and 0.3% knew someone at home who tested positive for COVID-19, which means that respondents knew up to seven students in their class who had COVID-19 (some may have known the same student) and three shared their home with someone with COVID-19.

### Effect on student mental health

**Fig. 2:**
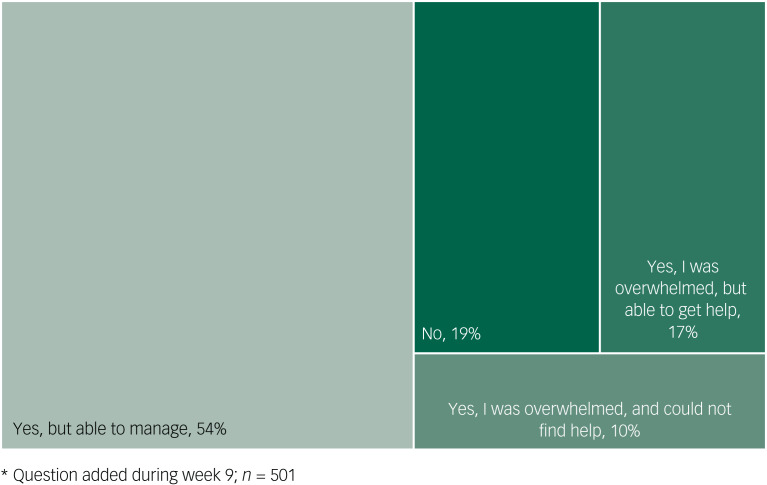
Treemap of responses to the question “Has the COVID-19 pandemic affected your emotional wellbeing?”*.

As a first step in understanding how students assessed the impact of the pandemic on their emotional well-being and their ability to get help, we summarise their responses through a treemap, which shows that although the majority was either unaffected or able to manage, important fractions were emotionally overwhelmed and unable to get help (Fig. 2).

Our exposure variable could take on three positive values: knowing someone outside of Canada who tested positive, knowing someone in Canada but not in Vancouver who tested positive, and knowing someone in Vancouver who tested positive (respondents were asked to choose the option that was geographically closest to Vancouver). A follow-up question asked about whether they lived together or were in the same class with the person with COVID-19 (see Supplementary Appendix 1, Box1). Table 1 shows that ‘knowing someone who tested positive for COVID-19 in Vancouver’ was significantly associated with increased symptoms. Of respondents who knew local people with COVID-19, the ratio of people with symptoms of anxiety or depression versus people without symptoms was >10:1. Given that this proximity seemed to capture the most significant effect, we dichotomised this variable (‘knowing someone in Vancouver who tested positive for COVID-19’) for all the linear regression models presented below.

Table 2 shows the coefficients, s.e., and *P*-values for the variables with statistically significant estimates in linear regression models, where the outcome variable is an indicator that the respondent had symptoms of anxiety or depression over the past 30 days. We present estimates for three types of regressions: univariable (variable × variable) in the leftmost panel, multivariable without interactions in the middle panel, and multivariable with interactions in the rightmost panel, with 30-day anxiety or depression symptoms as an outcome. Estimates for all coefficients (statistically significant or otherwise) are in Supplementary Appendix 2. In these linear models, the coefficients give the estimated change in the probability (in percentage points) of experiencing anxiety or depression associated with a unit change in a covariate. In the leftmost panel, we see that in the univariable models, knowing anyone in Vancouver who tested positive for COVID-19 is associated with an increase in the probability of any symptoms of 14 percentage points (s.e. 0.03, *P* ≤ 0.001).

**Table 2:** Univariable, multivariable and multivariable with interaction linear regressions for symptoms of anxiety or depression^a^

	Univariable^b^	Multivariable, no interactions^c^	Multivariable with interactions^c^
Predictor	Coefficient (robust s.e.)	Coefficient (robust s.e.)	Coefficient (robust s.e.)
Gender			
Male (reference group)	Reference	Reference	Reference
Female	0.11 (0.02)***	0.07 (0.03)**	0.08 (0.03)**
Other	0.29 (0.02)***	0.18 (0.04)***	0.19 (0.04)***
Week of completion			
1 (reference group)	Reference	Reference	Reference
2	−0.13 (0.07)	−0.13 (0.07)	−0.13 (0.07)
3	−0.05 (0.07)	−0.00 (0.07)	−0.00 (0.07)
4	−0.08 (0.06)	−0.06 (0.07)	−0.06 (0.07)
5	−0.10 (0.07)	−0.06 (0.07)	−0.06 (0.07)
6	−0.18 (0.07)**	−0.09 (0.07)	−0.10 (0.07)
7	−0.10 (0.07)	−0.07 (0.07)	−0.06 (0.07)
8	−0.10 (0.06)	−0.07 (0.07)	−0.06 (0.07)
9	−0.09 (0.07)	−0.06 (0.07)	−0.06 (0.07)
10	−0.06 (0.06)	−0.01 (0.07)	−0.03 (0.07)
11	−0.07 (0.07)	−0.02 (0.06)	−0.02 (0.07)
12	−0.06 (0.06)	−0.01 (0.07)	−0.02 (0.07)
13	−0.09 (0.07)	−0.10 (0.07)	−0.01 (0.07)
Survey type			
Initial survey (reference group)	Reference	Reference	Reference
Hard to reach with telephone	−0.07 (0.03)*	−0.04 (0.04)	−0.04 (0.04)
Hard to reach without telephone	−0.03 (0.06)	0.01 (0.06)	0.01 (0.06)
History of depression	0.22 (0.02)***	0.15 (0.03)***	0.16 (0.03)***
History of anxiety	0.29 (0.03)***	0.21 (0.04)***	0.21 (0.04)***
Knows someone who tested positive for COVID-19 in Vancouver?	0.14 (0.03)***	0.11 (0.04)	0.21 (0.22)
Housing type			
Living with parents or other relatives (reference group)	Reference	Reference	Reference
Living in own home or apartment (owned or rented)	−0.04 (0.03)	−0.07 (0.03)*	−0.08 (0.03)*
Living in a university owned or operated residence or fraternity	−0.06 (0.03)	−0.05 (0.04)	−0.05 (0.04)
Living in a shared house, apartment or flat	0.02 (0.03)	−0.02 (0.04)	−0.02 (0.04)
Other	−0.05 (0.11)	−0.11 (0.10)	−0.10 (0.10)

a. Only predictors with significant results shown, see complete table with nonsignificant predictors in Supplementary Appendix 2.

b. *n* = 1388.

c. *n* = 1188 (only participants with no missing data were included).

* *P* ≤ 0.05, **P ≤ 0.01, ***P ≤ 0.001.

Tables 3 and 4 show analogous models whose dependent variable is anxiety or depression separately. The middle panels present multivariable regression estimates, without interaction terms. These estimates indicate that the exposure is associated with an increase in the probability of having anxiety of 11 percentage points (Table 3; s.e. 0.05, *P* = 0.03), but is not significantly associated with depression (Table 4).

**Table 3:** Univariable, multivariable and multivariable with interaction linear regressions for symptoms of anxiety^a^

Predictor	Univariable^b^	Multivariable, no interactions^c^	Multivariable with interactions^c^
	Coefficient (robust s.e.)	Coefficient (robust s.e.)	Coefficient (robust s.e.)
Gender			
Male (reference group)	Reference	Reference	Reference
Female	0.14 (0.03)***	0.11 (0.03)***	0.11 (0.03)***
Other	0.27 (0.08)***	0.15 (0.09)	0.15 (0.09)
Week of completion			
1 (reference group)	Reference	Reference	Reference
2	−0.13 (0.07)	−0.13 (0.07)	−0.13 (0.07)
3	−0.05 (0.07)	−0.00 (0.07)	−0.00 (0.07)
4	−0.08 (0.06)	−0.06 (0.07)	−0.06 (0.07)
5	−0.10 (0.07)	−0.06 (0.07)	−0.06 (0.07)
6	−0.18 (0.07)**	−0.09 (0.07)	−0.10 (0.07)
7	−0.10 (0.07)	−0.07 (0.07)	−0.06 (0.07)
8	−0.10 (0.06)	−0.07 (0.07)	−0.06 (0.07)
9	−0.09 (0.07)	−0.06 (0.07)	−0.06 (0.07)
10	−0.06 (0.06)	−0.01 (0.07)	−0.03 (0.07)
11	−0.07 (0.07)	−0.02 (0.06)	−0.02 (0.07)
12	−0.06 (0.06)	−0.01 (0.07)	−0.02 (0.07)
13	−0.09 (0.07)	−0.10 (0.07)	−0.01 (0.07)
Survey type			
Initial survey (reference group)	Reference	Reference	Reference
Hard to reach with telephone	−0.10 (0.04)**	−0.05 (0.04)	−0.05 (0.04)
Hard to reach without telephone	−0.05 (0.07)	−0.02 (0.06)	−0.02 (0.07)
History of depression	0.24 (0.03)***	0.15 (0.03)***	0.15 (0.03)***
History of anxiety	0.34 (0.03)***	0.26 (0.04)***	0.27 (0.04)***
Knows someone in Vancouver who tested positive for COVID-19	0.13 (0.05)**	0.11 (0.05)*	0.39 (0.26)
Ethnicity			
White (reference group)	Reference	Reference	Reference
First Nations, Inuit or Metis	0.01 (0.08)	0.01 (0.07)	−0.01 (0.08)
Chinese	−0.06 (0.03)*	−0.03 (0.03)	−0.02 (0.04)
Non-Indigenous/Chinese visible minority	−0.03 (0.03)	−0.01 (0.03)	−0.01 (0.03)
Gender×knowing someone who tested positive for COVID-19 in Vancouver interaction			
Male×yes (reference group)	Reference	Reference	Reference
Female×yes	–	–	−0.20 (0.09)*

a. Only significant results shown, see complete table in Supplementary Appendix 2.

b. *n* = 1388.

c. *n* = 1188 (only participants with no missing data were included).

* *P* ≤ 0.05, **P ≤ 0.01, ***P ≤ 0.001.

**Table 4:** Univariable, multivariable and multivariable with interaction linear regressions for symptoms of depression^a^

Predictor	Univariable^b^	Multivariable no interactions^c^	Multivariable with interactions^c^
	Coefficient (robust s.e.)	Coefficient (robust s.e.)	Coefficient (robust s.e.)
Gender			
Male (reference group)	Reference	Reference	Reference
Female	0.12 (0.03)***	0.11 (0.03)***	0.11 (0.03)***
Other	0.30 (0.09)***	0.18 (0.09)	0.18 (0.09)
Age, years			
≤18 (reference group)	Reference	Reference	Reference
19	0.04 (0.05)	−0.02 (0.07)	−0.01 (0.07)
20	−0.04 (0.06)	−0.15 (0.08)	−0.14 (0.08)
21	−0.02 (0.06)	−0.17* (0.09)	−0.16 (0.09)
22	0.06 (0.06)	−0.07 (0.09)	−0.06 (0.09)
23	−0.04 (0.06)	−0.16 (0.09)	−0.15 (0.09)
24	−0.00 (0.07)	−0.06 (0.10)	−0.04 (0.10)
≥25	−0.07 (0.05)	−0.06 (0.09)	−0.05 (0.09)
Week of completion			
1 (reference group)	Reference	Reference	Reference
2	−0.05 (0.08)	0.01 (0.07)	0.01 (0.08)
3	−0.03 (0.07)	0.00 (0.07)	−0.00 (0.08)
4	−0.09 (0.07)	−0.05 (0.07)	−0.06 (0.07)
5	−0.14 (0.08)	−0.10 (0.08)	−0.10 (0.08)
6	−0.16 (0.07)*	−0.06 (0.07)	−0.11 (0.08)
7	−0.10 (0.07)	−0.09 (0.07)	−0.05 (0.07)
8	−0.12 (0.07)	−0.04 (0.08)	−0.09 (0.08)
9	−0.07 (0.07)	−0.01 (0.08)	−0.02 (0.08)
10	−0.09 (0.07)	0.03 (0.08)	−0.04 (0.08)
11	−0.05 (0.07)	0.00 (0.08)	0.02 (0.08)
12	−0.04 (0.07)	0.00 (0.07)	−0.01 (0.08)
13	−0.05 (0.07)	−0.03 (0.08)	−0.02 (0.08)
Survey type			
Initial survey (reference group)	Reference	Reference	Reference
Hard to reach with telephone	−0.06 (0.04)	−0.04 (0.04)	−0.04 (0.04)
Hard to reach without telephone	−0.04 (0.07)	0.02 (0.07)	0.02 (0.07)
History of depression	0.28 (0.03)***	0.23 (0.03)***	0.23 (0.03)***
History of anxiety	0.30 (0.03)***	0.17 (0.04)***	0.19 (0.04)***
Knows someone who tested positive for COVID-19 in Vancouver	0.11 (0.05)*	0.06 (0.06)	0.33 (0.22)
Student type			
First-year undergraduate (reference group)	Reference	Reference	Reference
Second-year undergraduate	0.04 (0.05)	0.08 (0.06)	0.08 (0.06)
Third-year undergraduate	0.02 (0.05)	0.10 (0.07)	0.09 (0.07)
Fourth-year undergraduate	0.04 (0.05)	0.08 (0.08)	0.07 (0.08)
Graduate	−0.05 (0.04)	−0.02 (0.08)	−0.02 (0.08)
Other	−0.17 (0.06)**	−0.09 (0.09)	−0.08 (0.09)
Ethnicity			
White (reference group)	Reference	Reference	Reference
First Nations, Inuit or Metis	0.05 (0.09)	0.02 (0.08)	0.02 (0.09)
Chinese	−0.01 (0.03)	−0.02 (0.04)	−0.01 (0.04)
Non-Indigenous/Chinese visible minority	0.08 (0.03)*	0.07 (0.03)*	0.08 (0.04)*
Housing type			
Living with parents or other relatives (reference group)	Reference	Reference	Reference
Living in own home or apartment (owned or rented)	−0.10 (0.03)**	−0.12 (0.04)**	−0.13 (0.04)
Living in a university owned or operated residence or fraternity	−0.09 (0.04)*	−0.08 (0.04)	−0.08 (0.04)
Living in a shared house, apartment or flat	−0.04 (0.04)	−0.05 (0.04)	−0.06 (0.04)
Other	−0.11 (0.13)	−0.18 (0.13)	−0.19 (0.13)

a. Only significant results shown, see complete table in Supplementary Appendix 2.

b. *n* = 1388.

c. *n* = 1188 (only participants with no missing data were included).

* *P* ≤ 0.05, **P ≤ 0.01, ***P ≤ 0.001.

To obtain a more granular understanding of the exposure's association with adverse outcomes in different subgroups, in the rightmost panels of Tables 3 and 4 we added potentially relevant interactions with gender, lifetime symptoms of depression or anxiety and completion week. Only the interaction term for gender is statistically significant, and only in the model for anxiety (Table 3). This means that the exposure's association with negative outcomes for anxiety is statistically significantly moderated by gender: the statistically significant interaction for female gender and knowing someone who tested positive in Vancouver (coefficient −20, s.e. 0.09, *P* ≤ 0.05) indicates that the effect on anxiety is 20 percentage points larger for male students than for female students. This suggests that the increase of 11 percentage points seen in the model without interactions is an average over very large effects for male students and much smaller effects for female students.

## Discussion

Our study sought to track the social and geographic proximity of COVID-19 cases to campus; describe the emotional impact on students, and their ability to manage and access help; and study the association of ‘knowing someone with COVID-19 in Vancouver’ with the probability of having symptoms of anxiety and/or depression during the past 30 days.

Extrapolating these findings to the general student population (58 375), during the first wave of the pandemic, 3269 students may have known someone in Vancouver, 467 may have known someone in their course and 175 may have shared their home with a confirmed case.^47^

Knowing local COVID-19 cases was associated with significantly elevated probability of having 30-day symptoms of anxiety. This is in line with other studies that have also reported on the association between the social proximity of COVID-19 cases and increased symptoms of anxiety and depression.^12,48,49^ A history of anxiety or depression was also associated with increased probability of current anxiety or depression. This may be because of a heightened susceptibility to stress among individuals with pre-existing anxiety and depressive symptoms, resulting in relapses or worsening of already existing mental health conditions during COVID-19.^50^ This complements a previous study among confined university students in France, in which a history of psychiatric follow-up was significantly associated with at least one mental health outcome, including self-reported suicidal thoughts, severe distress, stress, anxiety and depression.^51^ Although female students have higher baseline symptoms of anxiety than male students in our sample, results from the interaction analysis suggest that the association of COVID-19 propinquity with anxiety is moderated by gender, and is substantially lower among female students. Previous studies have similarly reported an increased prevalence of mental health outcomes among women during the COVID-19 pandemic but, to our knowledge, this is the first study to also explore the association of psychological distress with geographic proximity of COVID-19.^31,51^ Taking this association into account, the significant increase in anxiety observed in our study was notably driven by male students, and despite having higher baseline anxiety, female students seem to be more resilient than male students in response to COVID-19 proximity.

Our results also indicate that although the vast majority of students (78%) had some symptoms of anxiety or depression during the past 30 days, an even larger majority (90%) were either unaffected or able to cope (with or without help) as the first wave swept through Vancouver. This seems to be a unique finding; other studies have reported only a minority of students being able to cope adequately with the stress related to the current situation, and majority of students exhibiting maladaptive coping behaviours such as denial and disengagement to help with stress and anxiety.^29,30^ Of note, these widespread symptoms are subthreshold and may very well be non-pathological emotional responses. However, it is also important to bear in mind that a smaller fraction of these respondents will also meet criteria for a disorder, and therefore need services. Importantly, 10% report being emotionally overwhelmed and unable to access help.

### Strengths and limitations

Several strengths and limitations must be considered. The weekly deployment was well-suited to track the social and geographic proximity of COVID-19 and its associations with student mental health. Further, a sampling strategy that included assertive outreach allowed us to obtain an adjusted response rate higher than in typical college student surveys.^52^ Despite these efforts, several unknown factors may determine non-response to our survey. With respect to the known factors, Supplementary Appendix 2, Table 4 shows the distribution of the stratifying variables in our respondents and in the general student population. With a few exceptions (such as 63% *v.* 56% female students, or 1.5% *v.* 4.5% non-degree students), the composition is remarkably consistent, lending credence to these findings. Another limitation is that the COVID-19 questions were included in week 4, potentially missing students that knew someone in Vancouver during weeks 1–3, although this seems unlikely given that only three cumulative cases were reported during that time in the Vancouver catchment area, and that during week 4 (after 13 cumulative cases), only one respondent knew someone. Also, this analysis does not focus on the many stressors associated with COVID-19 that are not captured by the proximity of cases, such as physical distancing, policies with respect to grades and examinations and general disruption of life among others; these variables can also be expected to influence mental symptoms. However, our inclusion of dummy variables representing each week the survey was administered partially deals with time-varying, community-level determinants. Another limitation is that our analysis focuses on a subset of symptoms assessed by the WHO WMH-CIDI screening questions, and does not capture other potential effects of COVID-19 propinquity, such as substance use or syndromes meeting crtiteria for mental disorders. It is also important to note that the cross-sectional data examined in this study prevents us from making causal claims. Finally, these data were collected in the specific context of Vancouver, Canada, which is a high-income setting with a well-funded and functional health system.

In conclusion, despite these limitations, our results may offer important information to decision makers. The proximity of COVID-19 cases to campus is associated with increased likelihood of anxiety, but not with increased likelihood of depressive symptoms. The association of our exposure (without considering its interaction with gender) suggests an 11-percentage-point overall increase in the probability of any anxiety. Given that the 30-day prevalence of anxiety symptoms in our sample was 71%, this means an increase of 15% in the probability of anxiety (11/71 = 15).

Our estimates including interaction terms suggest that the overall association of anxiety is moderated by gender. The interaction term of female gender with the exposure shows that the response of female students is 20 percentage points lower than that of male students; therefore, male students are much more likely to suffer these symptoms in response to propinquity of COVID-19, and the overall association is likely dominated by their anxiety responses. It should also be noted that there is no moderation for lifetime symptoms: both those with lifetime symptoms and those without may be equally affected by propinquity of COVID-19.

These findings may also have policy implications. As additional waves of the pandemic take place, administrators should take notice that university students seem resilient, with the vast majority able to manage well or access help. As expected, subthreshold symptoms of anxiety and depression are extremely common at baseline, but the probability of depressive symptoms did not increase as COVID-19 disseminated, and the increase in anxiety seemed notably driven by male students. From a mental health perspective, the accrued evidence can guide a flexible approach that allows for partial re-opening of campuses with ongoing monitoring and adjustments, leveraging proven mitigation strategies with the ultimate goal of minimising the risk of infection, protecting and supporting the most vulnerable, and keeping overarching restrictions at a minimum.

## Data Availability

The data that support the findings of this study are available on request from the corresponding author, D.V. The data are not publicly available due to their containing information that could compromise the privacy of research participants.

## Supplementary material

For supplementary material accompanying this paper visit http://dx.doi.org/10.1192/bjo.2021.24. click here to view supplementary material
