# Supplementary material for: Investigating the effect of COVID-19 dissemination on symptoms of anxiety and depression among university students
Source: BJPsych Open. 2021 Mar 19;7(2):e69. doi: 10.1192/bjo.2021.24 (PMC8058823; doi:10.1192/bjo.2021.24)
Supplement: Supplementary file 1 [file S2056472421000247sup001.docx]

**Appendix 1:**

**Box 1: COVID-19 survey questions**

**The BC Centre for Disease Control indicates the following symptoms are compatible with COVID-19: cough, sneezing, fever, sore throat, and difficulty breathing. Do you have (or had in the past month) any of these symptoms?**

|  | Yes, and I tested positive for COVID-19 |
| --- | --- |
|  | Yes, and I tested negative for COVID-19 |
|  | Yes, but I didn’t or couldn’t get tested for COVID-19 |
|  | No |

**Do you know anyone who tested positive for COVID-19 or you think tested positive for COVID-19?** *(If you know people in more than one place, choose the option closest to Vancouver.)*

|  | Yes, I know someone in Vancouver |
| --- | --- |
|  | Yes, I know someone in Canada, but not in Vancouver |
|  | Yes, I know someone but not in Canada |
|  | No |

**How many people do you know who tested positive or you think tested positive for COVID-19?**

[DROPDOWN LIST] 1, 2, 3, 4, 5, More than 5

**How do you know him/her/them?** *(Check all that apply.)*

|  | We live together (family or roommates) |
| --- | --- |
|  | We are in the same class |
|  | We are friends or family but not living together |
|  | We are acquaintances |

**Does anyone in your residence/house/apartment have symptoms such as a cough, sneezing, fever, sore throat, or difficulty breathing?**

|  | Yes, but they tested negative for COVID-19 |
| --- | --- |
|  | Yes, and I am not aware of whether they got tested |
|  | No |

**Does anyone in your classes have symptoms such as a cough, sneezing, fever, sore throat, or difficulty breathing?**

|  | Yes, but they tested negative for COVID-19 |
| --- | --- |
|  | Yes, and I am not aware of whether they got tested |
|  | No |

**Has the COVID-19 pandemic affected your emotional wellbeing?**

|  | No |
| --- | --- |
|  | Yes, but I was able to manage it |
|  | Yes, I was/am overwhelmed and couldn’t/can’t find help |
|  | Yes, I was/am overwhelmed but I was/am able to get help |

**Box 2: WHO-CIDI 30-day screening scales for depression and anxiety**

**In the past 30 days, how often did you have each of the following experiences?**

|  | **All or almost all the time** | **Most of**  **the time** | **Some of the time** | **A little of the time** | **None of the time** |
| --- | --- | --- | --- | --- | --- |
| 1. Feel sad or depressed? |  |  |  |  |  |
| 1. Feel discouraged about how things were going in your life? |  |  |  |  |  |
| 1. Take little or no interest or pleasure in things? |  |  |  |  |  |
| 1. Feel down on yourself, no good, or worthless? |  |  |  |  |  |

**In the past 30 days, how often did you have each of the following experiences?**

|  | **All or almost all the time** | **Most of**  **the time** | **Some of the time** | **A little of the time** | **None of the time** |
| --- | --- | --- | --- | --- | --- |
| 1. Feel worried or anxious? |  |  |  |  |  |
| 1. Worry about a number of different things in your life, such as your work, family, health, or finances? |  |  |  |  |  |
| 1. Feel more worried than other people in your same situation? |  |  |  |  |  |
| 1. Worry excessively or too much? |  |  |  |  |  |

**Box 3: Survey protocol**

Day 0: An invitation email is sent to a stratified random sample of 350 students.

Day 3: A reminder email is sent to non-completers.

Day 7: A second reminder email is sent to non-completers.

Day 10: The initial survey closes and 70 students who do not start the survey are randomly selected for follow-up. They receive follow-up in one of two ways:

1. If a valid phone number is on file- an email is sent to the student informing them of their selection for follow-up and notifying them that they will be called the following day.
2. If no valid phone number is on file- a personal email is sent from the Principal Investigator informing them of their selection for follow-up and explaining the importance of the survey and their response.

These emails both contain a link to a copy of the initial survey, designated the “hard-to-reach with phone survey” (a) or “hard-to-reach without phone survey” (b).

Day 11: The “hard-to-reach with phone” group (a) receive a call from the research team explaining the importance of the survey and their response. If the student does not answer the first call, a second call is made several hours later. If the student does not answer the second call, a voicemail is left. If no voicemail system is available, a text message is sent.

Day 17: The “hard-to-reach” surveys closes.

**Appendix 2:**

**Table 1: Study characteristics /sub populations by Anxiety, Depression, and Anxiety or depression (complete)**

| **Variable** | **Anxiety**  **n (%)** | | **Depression**  **n (%)** | | **Anxiety or depression**  **n (%)** | |
| --- | --- | --- | --- | --- | --- | --- |
|  | No | Yes | No | Yes | No | Yes |
| **Gender** |  | *** |  | *** |  | *** |
| Male | 178 (46.11) | 283 (29.92) | 220 (41.75) | 251 (30.02) | 137 (46.28) | 332 (31.38) |
| Female | 206 (53.37) | 648 (68.50) | 304 (57.69) | 570 (68.18) | 159 (53.72) | 709 (67.01) |
| Other | 2 (0.52) | 15 (1.59) | 3 (0.57) | 15 (1.79) | 0 (0.00) | 17 (1.61) |
| **Age** |  |  |  |  |  |  |
| 18 or younger | 37 (9.66) | 83 (8.79) | 47 (8.94) | 81 (9.74) | 29 (9.83) | 97 (9.20) |
| 19 | 50 (13.05) | 135 (14.30) | 62 (11.79) | 129 (15.50) | 36 (12.20) | 152 (14.42) |
| 20 | 44 (11.49) | 110 (11.65) | 64 (12.17) | 94 (11.30) | 38 (12.88) | 119 (11.29) |
| 21 | 50 (13.05) | 102 (10.81) | 59 (11.22) | 93 (11.18) | 36 (12.20) | 116 (11.01) |
| 22 | 28 (7.31) | 105 (11.12) | 41 (7.79) | 93 (11.18) | 23 (7.80) | 111 (10.53) |
| 23 | 26 (6.79) | 77 (8.16) | 43 (8.17) | 62 (7.45) | 20 (6.78) | 85 (8.06) |
| 24 | 18 (4.70) | 49 (5.19) | 25 (4.75) | 43 (5.17) | 13 (4.41) | 55 (5.22) |
| 25 or older | 130 (33.94) | 283 (29.98) | 185 (35.17) | 237 (28.49) | 100 (33.90) | 319 (30.27) |
| **Week of completion** |  |  |  |  |  |  |
| 1 | 14 (3.62) | 55 (5.81) | 21 (3.98) | 47 (5.62) | 9 (3.03) | 59 (5.58) |
| 2 | 26 (6.72) | 52 (5.50) | 30 (5.68) | 54 (6.46) | 20 (6.73) | 62 (5.86) |
| 3 | 26 (6.72) | 76 (8.03) | 36 (6.82) | 69 (8.25) | 20 (6.73) | 85 (8.03) |
| 4 | 33 (8.53) | 83 (8.77) | 47 (8.90) | 70 (8.37) | 26 (8.75) | 91 (8.60) |
| 5 | 28 (7.24) | 63 (6.66) | 42 (7.95) | 52 (6.22) | 23 (7.74) | 71 (6.71) |
| 6 | 42 (10.85) | 69 (7.29) | 52 (9.85) | 60 (7.18) | 34 (11.45) | 78 (7.37) |
| 7 | 32 (8.27) | 75 (7.93) | 45 (8.52) | 65 (7.78) | 24 (8.08) | 85 (8.03) |
| 8 | 36 (9.30) | 82 (8.67) | 51 (9.66) | 68 (8.13) | 33 (11.11) | 86 (8.13) |
| 9 | 30 (7.75) | 71 (7.51) | 40 (7.58) | 66 (7.89) | 22 (7.41) | 81 (7.66) |
| 10 | 28 (7.24) | 78 (8.25) | 43 (8.14) | 65 (7.78) | 21 (7.07) | 87 (8.22) |
| 11 | 29 (7.49) | 78 (8.25) | 39 (7.39) | 71 (8.49) | 20 (6.73) | 88 (8.32) |
| 12 | 31 (8.01) | 86 (9.09) | 42 (7.95) | 77 (9.21) | 24 (8.08) | 94 (8.88) |
| 13 | 32 (8.27) | 78 (8.25) | 40 (7.58) | 72 (8.61) | 21 (7.07) | 91 (8.60) |
| **Type of communication participant responded to** |  | * |  |  |  |  |
| Initial survey | 299 (77.26) | 791 (83.62) | 421 (79.73) | 697 (83.37) | 230 (77.44) | 879 (83.08) |
| Hard to reach with phone | 73 (18.86) | 124 (13.11) | 88 (16.67) | 112 (13.40) | 56 (18.86) | 144 (13.61) |
| Hard to reach without phone | 15 (3.88) | 31 (3.28) | 19 (3.60) | 27 (3.23) | 11 (3.70) | 35 (3.31) |
| **History of depression?** |  | *** |  | *** |  | *** |
| No | 244 (63.21) | 325 (34.50) | 320 (60.84) | 265 (31.81) | 200 (67.57) | 378 (35.86) |
| Yes | 142 (36.79) | 617 (65.50) | 206 (39.16) | 568 (68.19) | 96 (32.43) | 676 (64.14) |
| **History of anxiety?** |  | *** |  | *** |  | *** |
| No | 166 (42.89) | 132 (14.0) | 185 (35.04) | 117 (14.01) | 133 (44.78) | 166 (15.70) |
| Yes | 221 (57.11) | 813 (86.03) | 343 (64.96) | 718 (85.99) | 164 (55.22) | 891 (84.30) |
| **Knows someone who tested positive for COVID-19?** |  | * |  | * |  | * |
| No | 350 (90.44) | 811 (85.73) | 473 (89.58) | 716 (85.65) | 270 (90.91) | 912 (86.20) |
| Yes | 37 (9.56) | 135 (14.27) | 55 (10.42) | 120 (14.35) | 27 (9.09) | 146 (13.80) |
| **Knows someone in Vancouver who tested positive for COVID-19?** |  | * |  | * |  | ** |
| No | 375 (96.90) | 885 (93.55) | 507 (96.02) | 782 (93.54) | 291 (97.98) | 991 (93.67) |
| Yes | 12 (3.10) | 61 (6.45) | 21 (3.98) | 54 (6.46) | 6 (2.02) | 67 (6.33) |
| **Anyone with flu-like or respiratory symptoms living in their residence, house or apartment? n=990** |  |  |  |  |  |  |
| No | 271 (91.86) | 635 (91.37) | 372 (92.77) | 555 (90.98) | 208 (91.63) | 712 (91.63) |
| Yes | 24 (8.14) | 60 (8.63) | 29 (7.23) | 55 (9.02) | 19 (8.37) | 65 (8.37) |
| **Anyone with flu-like or respiratory symptoms in their classes? n=988** |  | * |  | * |  | * |
| No | 256 (87.07) | 564 (81.27) | 344 (86.00) | 492 (80.79) | 198 (87.61) | 633 (81.57) |
| Yes | 38 (12.93) | 130 (18.73) | 56 (14.00) | 117 (19.21) | 28 (12.39) | 143 (18.43) |
| **Type of student** |  |  |  | ** |  |  |
| 1^st^ year undergraduate | 66 (17.60) | 152 (16.78) | 87 (17.13) | 142 (17.71) | 49 (17.13) | 176 (17.32) |
| 2^nd^ year undergraduate | 56 (14.93) | 148 (16.34) | 70 (13.78) | 139 (17.33) | 41 (14.34) | 166 (16.34) |
| 3^rd^ year undergraduate | 64 (17.07) | 162 (17.88) | 82 (16.14) | 149 (18.58) | 50 (17.48) | 181 (17.81) |
| 4^th^ year undergraduate | 62 (16.53) | 153 (16.89) | 75 (14.76) | 143 (17.83) | 46 (16.08) | 172 (16.93) |
| Graduate | 91 (24.27) | 229 (25.28) | 140 (27.56) | 184 (22.94) | 72 (25.17) | 250 (24.61) |
| Other | 36 (9.60) | 62 (6.84) | 54 (10.63) | 45 (5.61) | 28 (9.79) | 71 (6.99) |
| **Ethnicity** |  |  |  | * |  |  |
| White | 120 (31.09) | 341 (36.08) | 192 (36.43) | 275 (32.93) | 96 (32.43) | 371 (35.10) |
| First Nations, Inuit or Metis | 8 (2.07) | 24 (2.54) | 12 (2.28) | 21 (2.51) | 5 (1.69) | 28 (2.65) |
| Chinese | 128 (33.16) | 269 (28.47) | 171 (32.45) | 233 (27.90) | 102 (34.46) | 298 (28.19) |
| Other minority | 130 (33.68) | 311 (32.91) | 152 (28.84) | 306 (36.65) | 93 (31.42) | 360 (34.06) |
| **International student?** |  |  |  |  |  |  |
| No | 239 (67.32) | 612 (69.78) | 325 (66.46) | 549 (71.21) | 183 (66.79) | 685 (69.97) |
| Yes | 116 (32.68) | 265 (30.22) | 164 (33.54) | 222 (28.79) | 91 (33.21) | 294 (30.03) |
| **Housing type** |  |  |  | * |  |  |
| With parents or other relatives | 103 (26.61) | 257 (27.17) | 121 (22.92) | 249 (29.78) | 73 (24.58) | 296 (27.98) |
| In their own home or apartment (owned  or rented) | 113 (29.20) | 285 (30.13) | 174 (32.95) | 232 (27.75) | 95 (31.99) | 307 (29.02) |
| In a university owned or operated  residence or fraternity | 101 (26.10) | 197 (20.82) | 128 (24.24) | 178 (21.29) | 78 (26.26) | 225 (21.27) |
| In a shared house, apartment or flat | 65 (16.80) | 196 (20.72) | 98 (18.56) | 168 (20.10) | 47 (15.82) | 218 (20.60) |
| Other | 5 (1.29) | 11 (1.16) | 7 (1.33) | 9 (1.08) | 4 (1.35) | 12 (1.13) |

*p≤0.05; **p≤0.01; ***p≤0.001; both Chi-squared and Fisher's exact tests were used where the cell counts are small

n = 1388

**Table 2: Univariate, multivariate and multivariate with interaction linear regressions for Anxiety or depression (complete)**

| **Predictor** | **Univariate φ** | **Multivariate no interactions γ** | **Multivariate with interactions γ** |
| --- | --- | --- | --- |
|  | Coefficient (robust SE) | Coefficient (robust SE) | Coefficient (robust SE) |
| **Gender** |  |  |  |
| Male (reference group) | Reference | Reference | Reference |
| Female | 0.11 (0.02) *** | 0.07 (0.03) ** | 0.08 (0.03) ** |
| Other | 0.29 (0.02) *** | 0.18 (0.04) *** | 0.19 (0.04) *** |
| **Age** |  |  |  |
| 18 or younger (reference group) | Reference | Reference | Reference |
| 19 | 0.04 (0.05) | 0.01 (0.06) | 0.00 (0.06) |
| 20 | -0.01 (0.05) | -0.05 (0.07) | -0.05 (0.07) |
| 21 | -0.01 (0.05) | -0.06 (0.07) | -0.07 (0.07) |
| 22 | 0.06 (0.05) | -0.01 (0.08) | -0.01 (0.08) |
| 23 | 0.04 (0.05) | -0.01 (0.08) | -0.01 (0.08) |
| 24 | 0.04 (0.06) | -0.02 (0.08) | -0.01 (0.08) |
| 25 or older | -0.01 (0.04) | -0.01 (0.07) | -0.02 (0.07) |
| **Week of completion** |  |  |  |
| 1 (reference group) | Reference | Reference | Reference |
| 2 | -0.13 (0.07) | -0.13 (0.07) | -0.13 (0.07) |
| 3 | -0.05 (0.07) | -0.00 (0.07) | -0.00 (0.07) |
| 4 | -0.08 (0.06) | -0.06 (0.07) | -0.06 (0.07) |
| 5 | -0.10 (0.07) | -0.06 (0.07) | -0.06 (0.07) |
| 6 | -0.18 (0.07) ** | -0.09 (0.07) | -0.10 (0.07) |
| 7 | -0.10 (0.07) | -0.07 (0.07) | -0.06 (0.07) |
| 8 | -0.10 (0.06) | -0.07 (0.07) | -0.06 (0.07) |
| 9 | -0.09 (0.07) | -0.06 (0.07) | -0.06 (0.07) |
| 10 | -0.06 (0.06) | -0.01 (0.07) | -0.03 (0.07) |
| 11 | -0.07 (0.07) | -0.02 (0.06) | -0.02 (0.07) |
| 12 | -0.06 (0.06) | -0.01 (0.07) | -0.02 (0.07) |
| 13 | -0.09 (0.07) | -0.10 (0.07) | -0.01 (0.07) |
| **Type of communication participant responded to** |  |  |  |
| Initial survey (reference group) | Reference | Reference | Reference |
| Hard to reach with phone | -0.07 (0.03) * | -0.04 (0.04) | -0.04 (0.04) |
| Hard to reach without phone | -0.03 (0.06) | 0.01 (0.06) | 0.01 (0.06) |
| **History of depression** | 0.22 (0.02) *** | 0.15 (0.03) *** | 0.16 (0.03) *** |
| **History of anxiety** | 0.29 (0.03) *** | 0.21 (0.04) *** | 0.21 (0.04) *** |
| **Knows someone who tested positive for COVID-19** | 0.07 (0.03) * | - | - |
| **Knows someone who tested positive for COVID-19 in Vancouver?** | 0.14 (0.03) *** | 0.11 (0.04) | 0.21 (0.22) |
| **Has someone in their residence with flu-like/respiratory symptoms** | -0.00 (0.05) | - | - |
| **Has someone in their classes with flu-like/respiratory symptoms** | 0.07 (0.03) * | - | - |
| **Student type** |  |  |  |
| 1^st^ year undergraduate  (reference group) | Reference | Reference | Reference |
| 2^nd^ year undergraduate | 0.02 (0.04) | 0.02 (0.05) | 0.03 (0.05) |
| 3^rd^ year undergraduate | 0.00 (0.04) | -0.00 (0.06) | 0.00 (0.06) |
| 4^th^ year undergraduate | 0.01 (0.04) | -0.02 (0.06) | -0.01 (0.06) |
| Graduate | -0.01 (0.04) | -0.03 (0.07) | -0.02 (0.07) |
| Other | -0.07 (0.05) | -0.06 (0.08) | -0.05 (0.08) |
| **Ethnicity** |  |  |  |
| White (reference group) | Reference | Reference | Reference |
| First Nations, Inuit or Metis | 0.05 (0.07) | 0.05 (0.06) | 0.05 (0.06) |
| Chinese | -0.05 (0.03) | -0.03 (0.03) | -0.03 (0.03) |
| Non-Indigenous/Chinese Visible Minority | 0.00 (0.03) | 0.01 (0.03) | 0.02 (0.03) |
| **International Student?** | -0.03 (0.03) | -0.02 (0.03) | -0.02 (0.03) |
| **Housing type** |  |  |  |
| Living with parents or other relatives (reference  group) | Reference | Reference | Reference |
| Living in own home or apartment (owned or  rented) | -0.04 (0.03) | -0.07 (0.03) * | -0.08 (0.03) * |
| Living in a university owned or operated residence  or fraternity | -0.06 (0.03) | -0.05 (0.04) | -0.05 (0.04) |
| Living in a shared house, apartment or flat | 0.02 (0.03) | -0.02 (0.04) | -0.02 (0.04) |
| Other | -0.05 (0.11) | -0.11 (0.10) | -0.10 (0.10) |
| **History of anxiety-Knowing someone who tested positive for COVID-19 in Vancouver interaction** |  |  |  |
| No-Yes (reference group) | Reference | Reference | Reference |
| Yes-Yes | - | - | 0.00 (0.12) |
| **History of depression-Knowing someone who tested positive for COVID-19 in Vancouver interaction** |  |  |  |
| No-Yes (reference group) | Reference | Reference | Reference |
| Yes-Yes | - | - | -0.09 (0.10) |
| **Gender-Knowing someone who tested positive for COVID-19 in Vancouver interaction** |  |  |  |
| Male-Yes (reference group) | Reference | Reference | Reference |
| Female-Yes | - | - | -0.11 (0.07) |
| **Completion week-Knowing someone who tested positive for COVID-19 in Vancouver interaction** |  |  |  |
| 4-Yes | - | - | 0.32 (0.21) |
| 6-Yes | - | - | 0.18 (0.15) |
| 7-Yes | - | - | -0.44 (0.35) |
| 8-Yes | - | - | -0.15 (0.26) |
| 9-Yes | - | - | 0.13 (0.17) |
| 10-Yes | - | - | 0.15 (0.16) |
| 11-Yes | - | - | -0.02 (0.15) |
| 12-Yes | - | - | 0.16 (0.16) |
| **Ethnicity-Knowing someone who tested positive for COVID-19 in Vancouver interaction** |  |  |  |
| White-Yes (reference group) | Reference | Reference | Reference |
| First Nations, Inuit or Metis-Yes | - | - | -0.03 (0.09) |
| Chinese-Yes | - | - | -0.13 (0.11) |
| Other minority-Yes | - | - | -0.06 (0.09) |

*p≤0.05; **p≤0.01; ***p≤0.001

**φ** n = 1388; **γ** n = 1188 (only participants with no missing data were included)

Other gender and weeks 1, 2, 3, 5 and 13-Knowing someone who tested positive for COVID-19 in Vancouver interaction terms were omitted due to collinearity

**Table 3a: Univariate, multivariate and multivariate with interaction linear regressions for Anxiety symptoms (complete)**

| **Predictor** | **Univariate φ** | **Multivariate no interactions γ** | **Multivariate with interactions γ** |
| --- | --- | --- | --- |
|  | Coefficient (robust SE) | Coefficient (robust SE) | Coefficient (robust SE) |
| **Gender** |  |  |  |
| Male (reference group) | Reference | Reference | Reference |
| Female | 0.14 (0.03) *** | 0.11 (0.03) *** | 0.11 (0.03) *** |
| Other | 0.27 (0.08) *** | 0.15 (0.09) | 0.15 (0.09) |
| **Age** |  |  |  |
| 18 or younger (reference group) | Reference | Reference | Reference |
| 19 | 0.04 (0.05) | 0.02 (0.06) | 0.02 (0.06) |
| 20 | 0.02 (0.06) | -0.02 (0.07) | -0.02 (0.08) |
| 21 | -0.02 (0.06) | -0.09 (0.08) | -0.10 (0.08) |
| 22 | 0.10 (0.06) | 0.01 (0.08) | 0.00 (0.08) |
| 23 | 0.06 (0.06) | -0.03 (0.08) | -0.04 (0.09) |
| 24 | 0.04 (0.07) | -0.05 (0.09) | -0.05 (0.09) |
| 25 or older | -0.01(0.05) | -0.06 (0.08) | -0.07 (0.08) |
| **Week of completion** |  |  |  |
| 1 (reference group) | Reference | Reference | Reference |
| 2 | -0.13 (0.07) | -0.13 (0.07) | -0.13 (0.07) |
| 3 | -0.05 (0.07) | -0.00 (0.07) | -0.00 (0.07) |
| 4 | -0.08 (0.06) | -0.06 (0.07) | -0.06 (0.07) |
| 5 | -0.10 (0.07) | -0.06 (0.07) | -0.06 (0.07) |
| 6 | -0.18 (0.07) ** | -0.09 (0.07) | -0.10 (0.07) |
| 7 | -0.10 (0.07) | -0.07 (0.07) | -0.06 (0.07) |
| 8 | -0.10 (0.06) | -0.07 (0.07) | -0.06 (0.07) |
| 9 | -0.09 (0.07) | -0.06 (0.07) | -0.06 (0.07) |
| 10 | -0.06 (0.06) | -0.01 (0.07) | -0.03 (0.07) |
| 11 | -0.07 (0.07) | -0.02 (0.06) | -0.02 (0.07) |
| 12 | -0.06 (0.06) | -0.01 (0.07) | -0.02 (0.07) |
| 13 | -0.09 (0.07) | -0.10 (0.07) | -0.01 (0.07) |
| **Type of communication participant responded to** |  |  |  |
| Initial survey (reference group) | Reference | Reference | Reference |
| Hard to reach with phone | -0.10 (0.04) ** | -0.05(0.04) | -0.05 (0.04) |
| Hard to reach without phone | -0.05 (0.07) | -0.02 (0.06) | -0.02 (0.07) |
| **History of depression** | 0.24 (0.03) *** | 0.15 (0.03) *** | 0.15 (0.03) *** |
| **History of anxiety** | 0.34 (0.03) *** | 0.26 (0.04) *** | 0.27 (0.04) *** |
| **Knows someone who tested positive for COVID-19** | 0.09 (0.03) * | - | - |
| **Knows someone in Vancouver who tested positive for COVID-19** | 0.13 (0.05) ** | 0.11 (0.05) * | 0.39 (0.26) |
| **Has someone in their residence with flu like/respiratory symptoms** | 0.01 (0.05) | - | - |
| **Has someone in their classes with flu-like/respiratory symptoms** | 0.09 (0.04) * | - | - |
| **Student type** |  |  |  |
| 1^st^ year undergraduate  (reference group) | Reference | Reference | Reference |
| 2^nd^ year undergraduate | 0.03 (0.04) | -0.01 (0.05) | -0.00 (0.06) |
| 3^rd^ year undergraduate | 0.02 (0.04) | 0.00 (0.06) | 0.00 (0.06) |
| 4^th^ year undergraduate | 0.01 (0.04) | -0.01 (0.07) | -0.00 (0.07) |
| Graduate | 0.02 (0.04) | 0.02 (0.07) | 0.03 (0.07) |
| Other | -0.06 (0.06) | 0.05 (0.08) | -0.04 (0.09) |
| **Ethnicity** |  |  |  |
| White (reference group) | Reference | Reference | Reference |
| First Nations, Inuit or Metis | 0.01 (0.08) | 0.01 (0.07) | -0.01 (0.08) |
| Chinese | -0.06 (0.03) * | -0.03 (0.03) | -0.02 (0.04) |
| Non-Indigenous/Chinese Visible Minority | -0.03 (0.03) | -0.01 (0.03) | -0.01 (0.03) |
| **International Student** | -0.02 (0.03) | -0.02 (0.03) | -0.02 (0.03) |
| **Housing type** |  |  |  |
| Living with parents or other relatives (reference  group) | Reference | Reference | Reference |
| Living in own home or apartment (owned or  rented) | 0.00 (0.03) | -0.02 (0.04) | -0.02 (0.04) |
| Living in a university owned or operated residence  or fraternity | -0.05 (0.04) | -0.03 (0.04) | -0.03 (0.04) |
| Living in a shared house, apartment or flat | 0.04 (0.04) | 0.00 (0.04) | -0.01 (0.04) |
| Other | -0.03 (0.12) | -0.07 (0.11) | -0.06 (0.11) |
| **History of anxiety-Knowing someone who tested positive for COVID-19 in Vancouver interaction** |  |  |  |
| No-Yes (reference group) | Reference | Reference | Reference |
| Yes-Yes | - | - | 0.01 (0.15) |
| **History of depression-Knowing someone who tested positive for COVID-19 in Vancouver interaction** |  |  |  |
| No-Yes (reference group) | Reference | Reference | Reference |
| Yes-Yes | - | - | -0.12 (0.12) |
| **Gender-Knowing someone who tested positive for COVID-19 in Vancouver interaction** |  |  |  |
| Male-Yes (reference group) | Reference | Reference | Reference |
| Female-Yes | - | - | -0.20 (0.09) * |
| **Completion week-Knowing someone who tested positive for COVID-19 in Vancouver interaction** |  |  |  |
| 4-Yes | - | - | -0.92 (0.24) *** |
| 6-Yes | - | - | 0.10 (0.16) |
| 7-Yes | - | - | -0.47 (0.33) |
| 8-Yes | - | - | -0.25 (0.27) |
| 9-Yes | - | - | -0.16 (0.23) |
| 10-Yes | - | - | 0.01 (0.17) |
| 11-Yes | - | - | -0.05 (0.16) |
| 12-Yes | - | - | 0.02 (0.19) |
| **Ethnicity-Knowing someone who tested positive for COVID-19 in Vancouver interaction** |  |  |  |
| White-Yes (reference group) | - | - | - |
| First Nations, Inuit or Metis-Yes | - | - | 0.08 (0.11) |
| Chinese-Yes | - | - | -0.06 (0.12) |
| Other minority-Yes | - | - | -0.08 (0.13) |

*p≤0.05; **p≤0.01; ***p≤0.001

**φ** n = 1388; **γ** n = 1188 (only participants with no missing data were included)

Other gender and weeks 1, 2, 3, 5 and 13-Knowing someone who tested positive for COVID-19 in Vancouver interaction terms were omitted due to collinearity

**Table 3b: Univariate, multivariate and multivariate with interaction linear regressions for Depression symptoms (complete)**

| **Predictor** | **Univariate φ** | **Multivariate no interactions γ** | **Multivariate with interactions γ** |
| --- | --- | --- | --- |
|  | Coefficient (robust SE) | Coefficient (robust SE) | Coefficient (robust SE) |
| **Gender** |  |  |  |
| Male (reference group) | Reference | Reference | Reference |
| Female | 0.12 (0.03) *** | 0.11 (0.03) *** | 0.11 (0.03) *** |
| Other | 0.30 (0.09) *** | 0.18 (0.09) | 0.18 (0.09) |
| **Age** |  |  |  |
| 18 or younger (reference group) | Reference | Reference | Reference |
| 19 | 0.04 (0.05) | -0.02 (0.07) | -0.01 (0.07) |
| 20 | -0.04 (0.06) | -0.15 (0.08) | -0.14 (0.08) |
| 21 | -0.02 (0.06) | -0.17* (0.09) | -0.16 (0.09) |
| 22 | 0.06 (0.06) | -0.07 (0.09) | -0.06 (0.09) |
| 23 | -0.04 (0.06) | -0.16 (0.09) | -0.15 (0.09) |
| 24 | -0.00 (0.07) | -0.06 (0.10) | -0.04 (0.10) |
| 25 or older | -0.07 (0.05) | -0.06 (0.09) | -0.05 (0.09) |
| **Week of completion** |  |  |  |
| 1 (reference group) | Reference | Reference | Reference |
| 2 | -0.05 (0.08) | 0.01 (0.07) | 0.01 (0.08) |
| 3 | -0.03 (0.07) | 0.00 (0.07) | -0.00 (0.08) |
| 4 | -0.09 (0.07) | -0.05 (0.07) | -0.06 (0.07) |
| 5 | -0.14 (0.08) | -0.10 (0.08) | -0.10 (0.08) |
| 6 | -0.16 (0.07) * | -0.06 (0.07) | -0.11 (0.08) |
| 7 | -0.10 (0.07) | -0.09 (0.07) | -0.05 (0.07) |
| 8 | -0.12 (0.07) | -0.04 (0.08) | -0.09 (0.08) |
| 9 | -0.07 (0.07) | -0.01 (0.08) | -0.02 (0.08) |
| 10 | -0.09 (0.07) | 0.03 (0.08) | -0.04 (0.08) |
| 11 | -0.05 (0.07) | 0.00 (0.08) | 0.02 (0.08) |
| 12 | -0.04 (0.07) | 0.00 (0.07) | -0.01 (0.08) |
| 13 | -0.05 (0.07) | -0.03 (0.08) | -0.02 (0.08) |
| **Type of communication participant responded to** |  |  |  |
| Initial survey (reference group) | Reference | Reference | Reference |
| Hard to reach with phone | -0.06 (0.04) | -0.04 (0.04) | -0.04 (0.04) |
| Hard to reach without phone | -0.04 (0.07) | 0.02 (0.07) | 0.02 (0.07) |
| **History of depression** | 0.28 (0.03) *** | 0.23 (0.03) *** | 0.23 (0.03) *** |
| **History of anxiety** | 0.30 (0.03) *** | 0.17 (0.04) *** | 0.19 (0.04) *** |
| **Knows someone who tested positive for COVID-19** | 0.08 (0.04) * | - | - |
| **Knows someone who tested positive for COVID-19 in Vancouver** | 0.11 (0.05) * | 0.06 (0.06) | 0.33 (0.22) |
| **Has someone in their residence with flu-like/respiratory symptoms** | 0.06 (0.05) | - | - |
| **Has someone in their classes with flu-like/respiratory symptoms** | 0.09 (0.04) * | - | - |
| **Student type** |  |  |  |
| 1^st^ year undergraduate (reference group) | Reference | Reference | Reference |
| 2^nd^ year undergraduate | 0.04 (0.05) | 0.08 (0.06) | 0.08 (0.06) |
| 3^rd^ year undergraduate | 0.02 (0.05) | 0.10 (0.07) | 0.09 (0.07) |
| 4^th^ year undergraduate | 0.04 (0.05) | 0.08 (0.08) | 0.07 (0.08) |
| Graduate | -0.05 (0.04) | -0.02 (0.08) | -0.02 (0.08) |
| Other | -0.17 (0.06) ** | -0.09 (0.09) | -0.08 (0.09) |
| **Ethnicity** |  |  |  |
| White (reference group) | Reference | Reference | Reference |
| First Nations, Inuit or Metis | 0.05 (0.09) | 0.02 (0.08) | 0.02 (0.09) |
| Chinese | -0.01 (0.03) | -0.02 (0.04) | -0.01 (0.04) |
| Non-Indigenous/Chinese Visible Minority | 0.08 (0.03) * | 0.07 (0.03) * | 0.08 (0.04) * |
| **International Student** | -0.05 (0.03) | -0.02 (0.03) | -0.03 (0.03) |
| **Housing type** |  |  |  |
| Living with parents or other relatives (reference  group) | Reference | Reference | Reference |
| Living in own home or apartment (owned or  rented) | -0.10 (0.03) ** | -0.12 (0.04) ** | -0.13 (0.04) |
| Living in a university owned or operated residence  or fraternity | -0.09 (0.04) * | -0.08 (0.04) | -0.08 (0.04) |
| Living in a shared house, apartment or flat | -0.04 (0.04) | -0.05 (0.04) | -0.06 (0.04) |
| Other | -0.11 (0.13) | -0.18 (0.13) | -0.19 (0.13) |
| **History of anxiety-Knowing someone who tested positive for COVID-19 in Vancouver interaction** |  |  |  |
| No-Yes (reference group) | Reference | Reference | Reference |
| Yes-Yes | - | - | -0.29 (0.17) |
| **History of depression-Knowing someone who tested positive for COVID-19 in Vancouver interaction** |  |  |  |
| No-Yes (reference group) | Reference | Reference | Reference |
| Yes-Yes | - | - | 0.08 (0.15) |
| **Gender-Knowing someone who tested positive for COVID-19 in Vancouver interaction** |  |  |  |
| Male-Yes (reference group) | Reference | Reference | Reference |
| Female-Yes | - | - | -0.04 (0.13) |
| **Completion week-Knowing someone who tested positive for COVID-19 in Vancouver interaction** |  |  |  |
| 4-Yes | - | - | 0.85 (0.26) *** |
| 6-Yes | - | - | 0.18 (0.33) |
| 7-Yes | - | - | -0.65 (0.23) ** |
| 8-Yes | - | - | -0.11 (0.33) |
| 9-Yes | - | - | -0.21 (0.24) |
| 10-Yes | - | - | 0.14 (0.19) |
| 11-Yes | - | - | 0.05 (0.19) |
| 12-Yes | - | - | 0.13 (0.20) |
| **Ethnicity-Knowing someone who tested positive for COVID-19 in Vancouver interaction** |  |  |  |
| White-Yes (reference group) | Reference | Reference | Reference |
| First Nations, Inuit or Metis-Yes | - | - | -0.10 (0.37) |
| Chinese-Yes | - | - | -0.16 (0.14) |
| Other minority-Yes | - | - | -0.16 (0.14) |

*p≤0.05; **p≤0.01; ***p≤0.001

**φ** n = 1388; **γ** n = 1188 (only participants with no missing data were included)

Other gender and weeks 1, 2, 3, 5 and 13-Knowing someone who tested positive for COVID-19 in Vancouver interaction terms were omitted due to collinearity

**Table 4: Respondent and population demographics**

|  | **Respondent Demographics** | **Population Demographics** |
| --- | --- | --- |
| **Gender** |  |  |
| Female | 63.4% | 55.9% |
| Male | 35.2% | 44.1% |
| Other | 1.3% | - |
| **Age** |  |  |
| 18 or younger | 9.2% | 10.8% |
| 19 | 14.2% | 12.2% |
| 20 | 11.9% | 12.2% |
| 21 | 11.8% | 12.6% |
| 22 | 10.0% | 11.2% |
| 23 | 7.6% | 7.9% |
| 24 | 4.8% | 5.4% |
| 25 or older | 30.5% | 27.7% |
| **Student Status** |  |  |
| Domestic | 69.0% | 72.0% |
| International | 31.0% | 28.0% |
| **Program** |  |  |
| Undergraduate | 69.6% | 72.5% |
| Graduate | 23.4% | 19.4% |
| Non-degree | 1.5% | 4.5% |
| Other | 5.6% | 3.6% |
| **Year of Study** |  |  |
| 1 | 23.9% | 24.1% |
| 2 | 22.3% | 22.1% |
| 3 | 24.8% | 26.9% |
| 4 | 28.9% | 26.9% |

**Box 1: Adjusted Response Rate: 49.6%**

Obtained following the guidelines for a weighted response rate for a 2-phase sampling procedure^^[[1]](#footnote-1)^^

Calculation with average response rates:

Initial survey                      = 27% of total population with a weight of 1.

Hard to reach with phone         = 53% with a 46% response rate

Hard to reach without phone          = 47% with a 13% response rate

1*0.272 +  .463*(.530(.728)) + .132*(.470.(0.728))= 0.496

1. The American Association for Public Opinion Research. Standard Definitions: Final Dispositions of Case Codes and Outcome Rates for Surveys. 9th Edition.; 2016. [↑](#footnote-ref-1)
